# Psychological Factors Linked to Intimate Partner Violence and Childhood Maltreatment: On Dissociation as a Possible Bridge Symptom

**DOI:** 10.1177/08862605231181377

**Published:** 2023-07-11

**Authors:** Annegret Krause-Utz, Romana Černáková, William Hoogenboom, Anna Schulze, Sarah Büttner, Zeynep Demirelli, Joanne Mouthaan, Charlotte C. van Schie, Nadia Garnefski, Vivian Kraaij

**Affiliations:** 1 Leiden University, The Netherlands; 2 Heidelberg University, Germany; 3 University of Wollongong, NSW, Australia

**Keywords:** childhood maltreatment, dissociation, domestic violence, intimate partner violence, trauma

## Abstract

Intimate partner violence (IPV) is a serious health concern, occurring worldwide in various forms and settings. Over the past years, multiple sources reported an increase of IPV globally, partly related to COVID-19 restrictions. Childhood maltreatment enhances the risk of IPV, possibly via alterations in emotion regulation, attachment, maladaptive core beliefs, dissociation, and psychopathological symptoms. However, studies investigating these associations simultaneously are still needed. This study aimed to investigate association between IPV, childhood maltreatment severity, maladaptive schemata (mistrust, alienation, enmeshment), attachment anxiety, social support, emotion regulation, dissociation, posttraumatic stress disorder (PTSD), and borderline personality disorder (BPD) symptoms. We further explored the complex interplay of all factors, accounting for their shared associations. An anonymous online survey was posted on international online platforms for people experiencing domestic violence and on research platforms. Regression analyses and graph-theoretical network analysis were used to explore associations between all variables. *N* = 434 participants (40% in treatment) completed the survey. IPV perpetration and victimization were highly correlated. Both were significantly associated with childhood maltreatment severity, early maladaptive schemata, dissociation, BPD features, and PTSD symptoms. When including all variables in one model, IPV was associated with dissociation, which indirectly linked it to childhood maltreatment experiences, PTSD symptoms, withdrawal, and self-blame. Our findings suggest that IPV perpetration and victimization often co-occur. Dissociation may be an important bridge symptom, linking IPV to childhood maltreatment experiences, PTSD symptoms, and maladaptive coping. Prospective studies are needed to corroborate these findings and to establish psychological mechanisms underlying IPV.

Intimate partner violence (IPV) is a serious societal concern, occurring worldwide in various forms and settings (Alfaro Quezada et al., 2020; Black et al., 2011). Expressions of IPV can be complex and multi-faceted, involving one or multiple forms of psychological, physical, and/or sexual violence. Previous research suggests that IPV is frequently reciprocal, that is, perpetration and victimization co-occur, creating complex dysfunctional relationship dynamics, which are hard to break (Whitaker et al., 2007). IPV can have detrimental consequences; it leads to various adverse health outcomes and increases the risk of suicide and homicide (Capaldi et al., 2012; Stith et al., 2004).

Over the past years, multiple sources have reported an increase of IPV globally, partly related to stay-at-home orders and other governmental restrictions that were put in place during the COVID-19 pandemic (Evans et al., 2020; Parrott et al., 2022; Pattojoshi et al., 2021; van Gelder et al., 2020). This alarming increase highlights the persistent need to detect risk factors and psychological correlates of IPV.

Representative surveys suggest that IPV affects both biological sexes equally, with approximately 15–35% of women and 20-25% of men reporting incidents of IPV in the United States (Breckenridge et al., 2019; Capaldi et al., 2012; Stith et al., 2004). At the same time, women seem to be more frequently affected by serious injuries and sexual IPV than men (12.4% vs. 2.1%, in Fanslow et al., 2022). Recent studies found a higher prevalence of IPV in minority groups (Basile et al., 2021; Campbell et al., 2023; Ummak et al., 2022) and in groups with lower socioeconomic status (Campbell et al., 2023; Cho, 2012). Other risk factors include life stress, lower parental education, and environments condoning partner violence (Capaldi et al., 2012; Stith et al., 2004; Yakubovich et al., 2018).

Moreover, it has been shown that a history of childhood maltreatment (abuse and neglect) is an important risk factor of IPV (Li et al., 2019). Prospective studies revealed particularly strong associations for physical abuse (Caetano et al., 2005; Cui et al., 2010; Stith et al., 2004; Widom et al., 2014). Several psychological mechanisms have been implicated in this cycle of violence, that is, alterations in core beliefs, emotion regulation, attachment formation, dissociation, and psychopathologies, such as posttraumatic stress disorder (PTSD) and borderline personality disorder (BPD) (Hébert et al., 2021). These psychological factors may partly account for the link between childhood maltreatment and IPV, although their complex interplay is not yet entirely understood.

Growing up in an abusive or violent family environment influences the way how people think about themselves and other people, for example, can lead to early maladaptive schemata, such as mistrust, alienation, and enmeshment (Young, 1990). Mistrust is the belief or expectation that others will hurt, abuse, or humiliate oneself. Alienation is the sense of being fundamentally different or isolated from others. Enmeshment involves a diminished sense of self and an over-involvement in the life of significant others (Young, 1990), which can lead to interpersonal dependency (Gomez, 2012; Hassija et al., 2018; Kivisto et al., 2015). A recent meta-analysis points to an important role of early maladaptive schemata in IPV, which may reinforce preexisting core beliefs (Pilkington et al., 2021).

Maladaptive schemata, such as mistrust, alienation, and enmeshment, often go hand in hand with an anxious attachment style. Early interactions with primary caregivers are important for attachment formation and severe abuse can lead to insecure (anxious, avoidant, preoccupied) attachment styles (Bowlby, 1958; Karatzias et al., 2021). Anxiously attached individuals are overly sensitive to their partner’s state, fear rejection, or abandonment and may find it harder to leave abusive relationships (Bonache et al., 2017; Kong et al., 2018; Lawson & Brossart, 2013; McKeown, 2014). Several, but not all previous studies, found significant associations between anxious attachment and IPV (see systematic review by Velotti et al., 2018). Other studies suggest that individuals who have repeatedly experienced interpersonal violence have more difficulties seeking and/or getting social support outside of their intimate relationship, for example, due to general mistrust and maladaptive coping styles, such as ignoring and social withdrawal (Collins & Feeney, 2004).

Childhood maltreatment can interfere with the development of adaptive emotion regulation. Coping strategies, such as self-blame, suppression, avoidance (ignoring), and withdrawal, are often acquired as survival strategies to escape distressing experiences in the short run (Gruhn & Compas, 2020). In the long run, these strategies may increase emotional vulnerability and enhance the risk of revictimization (Batten et al., 2001; Roth & Cohen, 1986; Scoglio et al., 2021). A lack of adaptive coping strategies may further contribute to impulsive aggression and enhance the risk that stressful conflicts escalate into violence.

In a similar manner, dissociative symptoms, such as de-personalization, de-realization, and memory fragmentation, may help to cope with overwhelming traumatic experiences (Dorahy et al., 2016; Vonderlin et al., 2018), while interfering with information processing (Krause-Utz et al., 2021; Zurbriggen & Freyd, 2004)). In prospective research (Noll et al., 2003; Zamir et al., 2018) individuals who experienced more severe dissociation after child sexual abuse were more often re-victimized in adult relationships. Dissociation can also lead to subjective disconnection from abusive or violent behaviors during IPV perpetration. This may be influenced by trauma-based emotional dysregulation and experienced as losing control, observing oneself from a wider distance while aggressing, and having blackouts. In the long run, violent acts occurring during dissociation may be disconnected from other identity parts and stored as separate memories, hindering adaptive problem-solving processes (Webermann & Brand, 2017; Webermann & Murphy, 2019).

Some of the aforementioned alterations may occur in the context of psychopathologies, such as PTSD and BPD. PTSD is a psychiatric disorder, characterized by symptoms of re-experiencing (e.g., through nightmares and intrusions), avoidance of traumatic reminders (thoughts, behaviors, places, or persons associated with the event), negative changes in thoughts and mood, increased arousal, and heightened sense of threat after a traumatic event. Repeated interpersonal violence has been associated with more severe and complex symptom presentations of PTSD, including disturbances in emotion dysregulation, identity, and relationships (Gilbar et al; 2019; Karatzias et al., 2021). PTSD symptoms of avoidance and emotional disengagement were linked to revictimization in recently abused women (Iverson et al., 2013). Other PTSD symptoms, that is, hypervigilance and increased irritability, have been implicated in IPV as well (Birkley et al., 2016; Flanagan et al., 2014; Iverson et al., 2013; Scoglio et al., 2021; Sullivan et al., 2005).

BPD is associated with higher rates of childhood abuse and neglect (Porter et al., 2020) and with more IPV. A recent meta-analysis by Collison and Lynam (2021) synthesized findings of 163 studies on different personality disorders (PDs) and found that antisocial PD and BPD were consistently linked to IPV. Numerous studies found that individuals with BPD show higher rates of IPV perpetration (e.g., González et al., 2016; Maneta et al., 2013; Peters et al., 2017; Spencer et al., 2019), while both forms of IPV have been associated with (subclinical) features of BPD (Krause-Utz et al., 2018, 2021).

All in all, previous studies established associations between IPV and childhood maltreatment, early maladaptive schemata (mistrust, alienation, and enmeshment), anxious attachment, low social support, emotion dysregulation, dissociation, and symptoms of PTSD and BPD. Empirical research further suggests that these psychological factors partly overlap and are intertwined with each other. Maladaptive schemata, anxious attachment, low social support, emotion dysregulation, dissociation, and symptoms of PTSD and BPD may both be precursors and consequences of IPV, thereby maintaining the cycle of violence.

To our knowledge, no study so far has investigated the aforementioned psychological variables simultaneously to account for their complex multivariate interplay. Psychological network analysis can be used to investigate interdependencies between complex psychological constructs that have mutual influence on each other (Borsboom & Cramer, 2013). Even when a clear rationale for separating relevant constructs into predictors and outcomes is missing, psychological network analysis can be used to explore unique associations, which are not explained by other variables. Thereby, so-called bridge symptoms can be detected that connect different clusters of variables. These bridge symptoms may be of particular relevance in a broader clinical context, being primary targets for prevention and intervention. A recent study that used data-driven network analysis already points to an important role of childhood emotional abuse, adulthood psychological distress, and social isolation in 198 pregnant Hispanic women experiencing IPV (Diestel et al., 2023).

The aim of the current study was to investigate associations between IPV and childhood maltreatment, early maladaptive schemata (mistrust, alienation, and enmeshment), anxious attachment, low social support, emotion dysregulation, dissociation, and symptoms of PTSD and BPD. Based on previous literature, we expected significant positive correlations between IPV and childhood trauma severity, early maladaptive schemata, anxious attachment, emotion dysregulation, dissociation, PTSD symptoms, and BPD features. We further expected a negative correlation with perceived social support.

Based on earlier research, we further hypothesized that the association between childhood maltreatment severity and IPV would be explained by early maladaptive schemata (Young, 1990), attachment anxiety (Bowlby, 1958), maladaptive coping (Batten et al., 2001; Roth & Cohen), dissociation (Vonderlin et al., 2018), and BPD symptoms (Krause-Utz et al., 2018; 2021).

Since we were interested in both the direct and indirect associations with IPV, we additionally calculated a partial correlation network to explore the complex interplay of all factors simultaneously, accounting for their shared associations. In an exploratory hypothesis-generating manner, we examined bridge symptoms that may link childhood maltreatment severity to IPV.

## Methods

### Participants

Participants were recruited through international mental health online platforms for people who experienced family and/or partner violence, after administrators had given permission to post the survey. To gain a broader sample, we additionally recruited participants through diverse social media (Facebook, Instagram, Twitter etc.) and via the university research platform. Inclusion criteria were being aged above 18, indicating sufficient English proficiency [defined as ability to understand the main points of clear standard input on familiar matters regularly encountered in work, school, and leisure], and having been either in a past or present long-term relationship, lasting at least 3 months. Out of 935 respondents initially interested in the survey, *n* = 445 completed the relevant questionnaires of the survey. To be able to perform all statistical analysis, respondents who did not complete the relevant scales needed to be excluded post hoc.

Demographics of the full sample are presented in Table 1. The mean age was 25 years (SD = 10.22); 55% (*n* = 244) identified as female, 29% (*n* = 128) as male, and 16% (*n* = 73) as other. Most respondents were European (*n* = 366, 82.2%); about 40% (*n* = 179) were currently in a relationship, with 37.1% (*n* = 165) being single. Approximately half of the participants finished secondary education (*n* = 251, 56.4%). Majority of the participants were currently enrolled in an educational system (*n* = 351, 78.9%), and *n* = 239 (54.1%) were currently employed.

**Table 1.** Sample Characteristics.

Demographics	*Age (mean ± st. dev.)*	25.29 ± 10.22
*Education*	Primary School Secondary School Bachelor Degree Master Degree PhD Vocational Training Other	*n =* 4 (0.9%) *n =*251(56.4%) *n =*106(23.8%) *n =* 53 (11.9%) *n =* 6 (1.3%) *n =* 13 (2.9%) *n =* 12 (2.7%)
*Relationship Status*	Single In a relationship Married Divorced Separated Widowed Shared household Other	*n =*165(37.1%) *n =*179(40.2%) *n =* 48 (10.8%) *n =* 3 (0.7%) *n =* 1 (0.2%) *n =* 2 (0.4%) *n =* 7 (1.6%) *n =* 4 (0.9%)
*Nationality*	European Asian North American South American Middle East Other	*n =*366 (82.2%) *n =* 16 (3.6%) *n =* 6 (1.3%) *n =* 5 (1.1%) *n =* 38 (8.5%) *n =* 14 (3.1%)
*Childhood maltreatment*	Overall Emotional Abuse	114 (26%) 67 (15%)
	Emotional Neglect	63 (14%)
	Physical Abuse	38 (9%)
	Physical Neglect	32 (7%)
	Sexual Abuse	38 (9%)

In this final sample, incidents of IPV perpetration and victimization were highly correlated (*r* *=* .87, *p* < .001). The majority of participants reported incidents of IPV perpetration and victimization within the same relationship, mostly psychological aggression (*n* *=* 349, 78%), followed by physical aggression (23%), sexual coercion (8%), and severe injury (1%). No significant correlations between IPV frequency and demographics were found (*p* > .05).

According to established cutoffs (Bernstein et al., 2003; Glaesmer et al., 2013), *n* *=* 114 (26%) participants reported at least moderate to severe childhood trauma, mostly emotional abuse and neglect, followed by physical and sexual abuse, and physical neglect. While 50 participants (11.2%) reported clinically relevant BPD features (Personality Assessment Inventory—Borderline Feature Scale (PAI-BOR total ≥ 37, Jackson & Trull, 2001), 50 participants (11.2%) reported clinically relevant trait dissociation (DES ≥ 30; Bernstein & Putnam, 1986).

A total of 173 participants (40%), who received psychotherapeutic treatment, were recruited through international mental health online platforms. This subsample comprised more women (χ²_(4)_ *=* 10.32, *p* *=* .035) and reported significantly more severe childhood maltreatment (*F*_(1,389_ *=* 26.45, *p* < .0001), dissociation (*F* *=* 17.55, *p* < .0001), anxious attachment (*F* *=* 12.48, *p* < .0001), withdrawal (*F* *=* 13.35, *p* < .0001), maladaptive cognitive coping (*F* *=* 22.03, *p* < .0001), more BPD features (*F* *=* 15.46, *p* < .0001), and PTSD symptoms (*F* *=* 18.07, *p* < .0001), but did not report frequent IPV (all *p* > .060).

After the start of the COVID-19 pandemic (March 2020) 139 participants (31%) were recruited. At the start of the pandemic, we added the following question to our survey: “Are you affected by the current events concerning the corona virus pandemic?” Participants who confirmed that they were affected by the pandemic (*n* *=* 34) reported significantly more IPV victimization (*F*_(1,471)_ *=* 4.79, *p* *=* .030; perpetration: *F*_(1,471)_ *=* 0.40, *p* *=* .528). This subsample comprised more men and non-European, while there were no other significant demographic or clinical differences (Appendix A).

### Materials

A detailed overview of all measures and their psychometric properties can be found in Table 2. Intimate partner violence was assessed with the Revised Conflict Tactics Scale (CTS-2; Straus et al., 1996). The CTS-2 is a well-established scale that includes 78 pairs of questions referring to the “self” as well as the partner. Next to “negotiation” (e.g., “showed my partner I cared even though we disagreed”), four subscales measured IPV in terms of “psychological aggression” (e.g., “shouted or yelled at my partner”), “physical assault” (e.g., “threw something at my partner that could hurt,” “twisted my partner’s arm”), and “sexual coercion” (e.g., “used force to make my partner have sex”). “Injury” measures injuries of the “partner” (*perpetration*) and the “self” (*victimization*). Items are answered on a six-point scale, indicating the frequency of the respective behavior (between “0 *=* never” and “6 *=* more than 20 times”) within the same relationship. The CTS-2 has shown strong psychometric properties, including good construct and good discriminant validity and internal consistency (Table 2). Other established scales were used to assess childhood trauma (Childhood Trauma Questionnaire (CTQ; Bernstein et al., 2003); early maladaptive schemata (Young Schema Questionnaire short form (YSQ-SF; Bach et al., 2017); perceived social support (Multidimensional Scale of Perceived Social Support (MSPSS; Zimet et al., 1988); dissociation (Dissociative Experiences Scale (DES; Bernstein & Putnam, 1986); attachment (Revised Adult Attachment Scale (R-AAS; Collins, 1996); BPD features (PAI-BOR; Jackson & Trull, 2001); PTSD symptoms (PTSD Check List—DSM-5, PCL-5; Blevins et al., 2015; Bovin et al., 2016; Wortmann et al., 2016). Emotion regulation was measured using the Behavioral Emotion Regulation Questionnaire (BERQ; Kraaij & Garnefski, 2019) as well as the subscales rumination and self-blame of the Cognitive Emotion Regulation Questionnaire, (CERQ; Garnefski & Kraaij, 2007). Higher scores represent stronger expressions.

**Table 2.** Overview of Measures.

Time-Frame	Construct	Scale (Authors, Year of Publication)	Subscales	Items and Item Format (Example Item)	Reported Psychometric Properties	Cronbach’s Alpha in Current Sample
Repeated incidents in the same relationship	IPV perpetration and victimization	Conflict Tactics Scale, revised (CTS-2) (Straus et al., 1996)	Psychological aggression, physical assault, sexual coercion, and injury	78-items (between “0 = never” and “6 = more than 20 times”)	Internal consistency between 0.79 and 0.95	α = .94
Retrospective recall of childhood experiences	Severity of childhood maltreatment (abuse and neglect)	Childhood Trauma Questionnaire short form (CTQ) (Bernstein et al., 2003)	Emotional abuse, physical abuse, sexual abuse, emotional neglect, and physical neglect	25-items (between “1 = never true” and “5 = very often true”)	Test-retest reliability ranging from 0.79 to 0.84, internal consistency between α = .66 and α = .92	Emotional abuse: α = .88, sexual abuse: α = .90, physical abuse: α = .87, and emotional neglect: α = .89
Trait measures	Early maladaptive schemata	Young Schema Questionnaire (YSQ) (Bach et al., 2017)	Mistrust, alienation, and enmeshment	12-items (between “1 = completely untrue of me” and “6 = describes me perfectly”)	Test-retest reliability >0.70, internal consistency between 0.70 and 0.97	Mistrust α = .89, alienation α = .75, and enmeshment α = .74
	Attachment insecurity	Revised Adult Attachment Scale (R-AAS) (Collins, 1996)	Anxiety, attachment closeness, and attachment dependence	18-items (between “1 = not at all characteristic of me” and “5 = very characteristic of me”	Between Cronbach’s α = .74 and α = .86	
	Trait dissociation	Dissociation Experience Scale (DES) (Bernstein & Putnam, 1986)	—	28-items (between “0 = never” and “100 = always”	Split-half reliability between 0.71 and 0.96, test-retest reliability 0.84, construct validity between 0.50 and 0.79, and Cronbach α = .94	α = .94
	Borderline personality disorder features	Personality Assessment Inventory—Borderline Feature Scale (PAI-BOR) (Jackson & Trull, 2001)	Affective instability, identity diffusion, impulsive self-harm, and negative relationships	24-items (between “0 = false” and “3 = very true”)	Internal consistency between α = .68 (negative relationships) and α = .78 (self-harm)	α = .71
State measures	Perceived social support in domains of family, friends, and significant other	Multidimensional Scale of Perceived Social Support (MSPSS) (Zimet et al., 1988)	Family, friends, and significant other	12-items (between “1 = very strongly disagree” and “7 = very strongly agree”	Reliability of subscales >0.85, moderate construct validity	Friends α = .93, family α = .92, and significant other α = .94
	Posttraumatic stress disorder symptoms over the course of the past month	Posttraumatic Stress Disorder Checklist-DSM-5 (PCL-5) (American Psychiatric Association, 2013)	Re-experiencing, avoidance, negative alterations in mood and cognition, hyperarousal	20-items (between “not at all” and “extremely”)	Test-retest reliability between 0.82 and 0.84, internal consistency between α = .91 and α = .96	α = .94
	Behavioral coping after a recent stressful event	Behavioral Emotion Regulation Strategies Inventory (BERQ) (Kraaij & Garnefski, 2019)	Seeking distraction, actively approaching, seeking social support, social withdrawal, ignoring	25-items (between “1 = almost never” and “5 = almost always”)	Test-retest reliability between 0.47 and 0.75, internal consistency between α = 86 and α = .93	α = .87
	Cognitive emotion regulation strategies after a recent stressful event	Cognitive Emotion Regulation Questionnaire (CERQ)	Self-blame, catastrophizing, rumination	36-items (between “1 = (almost) never” and “5 = (almost) always”	Cronbach’s α between α = .60 and α = .85	Self-blame α = .81, rumination α = .77, catastrophizing α = .71

### Procedure

The study was approved by the local Psychology Ethics Committee, in line with international standards of Helsinki. Data collection took place between March 2019 and April 2021. The survey could be assessed via a link and a QR code via the software Qualtrics (© 2015, Qualtrics, Provo, UT). Participants were first informed about background, aims, potential risks, reimbursement for study participation, and the right to end the survey at any point of time without any negative consequences. A disclaimer was included in the information letter, highlighting the sensitive and potentially distressing nature of some questions. Only after informed consent had been given and inclusion criteria had been indicated, the survey proceeded, otherwise, it was automatically terminated. First, demographic variables were assessed. Then, the psychometric scales were presented in randomized order. After completion, participants answered questions about psychotherapeutic treatment. At the end, participants were debriefed and asked whether they had been “[. . .] unable to answer one or more questions due to a lack of English proficiency” (YES responses led to post hoc exclusion from analysis). Participants were encouraged to contact the Principal Investigator (AKU), a trained therapist, in case of discomfort due to the intimate nature of the items. Five participants contacted the PI but did not require further interventions. Completion of the survey took approximately 40 minutes. Respondents had the opportunity to participate in a lottery (chance of winning 25 Euro vouchers). Psychology students could gain study credits.

### Data Analysis

Data obtained in Qualtrics was exported to IBM SPSS Statistics 27.0. Significance level was a priori determined as *p* ≤ .05 (2-tailed). Frequency of IPV was represented by the CTS-2 sum score of the “self” and “partner” items. Since incidents of IPV perpetration and victimization were highly correlated (*r* *=* 0.87, *p* < .001) and the majority of participants reported both perpetration and victimization within the same relationship, we decided to focus on overall IPV as primary outcome. For matters of completeness, analyses were also performed for victimization and perpetration separately (see Appendixes B and C). Other variables were represented by sum scores (AAS, CTQ, MSPSS, PAI-BOR, and PCL) or mean scores (BERQ, CERQ, DES, and YSQ). Prior to analyses, assumptions of linearity, normality of residuals, homoscedasticity, and independence of residuals were checked. Collinearity diagnostics indicated that multicollinearity was unlikely; all tolerance values were above 0.4 and variance inflation factors (VIF) below 2.4. For participants with a maximum of one missing item per (sub-)scale, missing values were replaced with the (sub-)scale mean score, and participants with more than one missing per (sub-)scale were excluded from the analyses. The sample size for the final analysis comprised *n* *=* 432 participants.

Underlying bivariate associations between all variables were examined using Pearson correlations. To reduce the number of comparisons, we included only the total or sum score of these scales, except for the BERQ, CERQ, and YSQ-SF, which do not have total or sum scores. To correct for multiple comparisons, a Bonferroni correction for the 16 different scales was applied (*p* < .05 / 16 *=* *p* ≤ .002).

To test our hypothesis, separate linear regression analyses were performed with overall IPV (CTS-2 scores) as outcome variable, followed by a multiple regression analysis including all scales. Since participants affected by the COVID-19 pandemic reported significantly more IPV victimization, we added a categorical variable (before vs. after start of pandemic) as statistical covariate in the regression analyses.

### Network Analysis

To explore “unique” associations with IPV that were not explained by shared associations with other variables, a partial correlation network was calculated. Within this graphical network, elements (“nodes”) and their associations among each other (“edges”) can be visualized. To describe the features of such networks, each node can be characterized by quantifying its connections to other nodes in the network by the network parameter “centrality.” Nodes with higher centrality indices have more and/or stronger connections within the network (Borsboom & Cramer, 2013). We included 17 nodes in our network: the sum score of CTS-2, CTQ, MSPSS, PCL, and PAI-BOR, as well as the mean score of the DES and BERQ subscales (seeking distraction, withdrawal, ignoring, seeking social support, and actively approaching), CERQ subscales (rumination and self-blame), the AAS attachment anxiety subscale, and the YSQ-SF subscales enmeshment, alienation, and mistrust.

We estimated a regularized mixed graphical model using the *mgm* function of the package *mgm* (Haslbeck & Waldorp, 2020) as implemented in the *bootnet* package in R (Epskamp et al., 2018). Hereby, the edges calculated via nodewise regressions can be interpreted like partial correlations. The mixed graphical model is regularized by the least absolute shrinkage and selection operator (LASSO) (Tibshirani, 1996) to avoid false positives. The parameter λ, which controls the strength of the penalty, was selected using the extended Bayesian information criterion (EBIC) (Foygel & Drton, 2010) with the hyper-parameter set to default (γ *=* .25). Node predictability was calculated using the residual *R*^2^ error value from mixed Gaussian model (MGM) estimation for each network using the *mgm* package (Haslbeck & Waldorp, 2020). Node centrality strength was calculated as the sum of all absolute edge weights connected to a given node. In order to check the stability of the network, the correlation stability coefficient for node strength was calculated via case-drop bootstrapping and accuracy of edge weights and centrality parameters were calculated via nonparametric bootstrapping procedure implemented in the R-package *bootnet* (Epskamp et al., 2018). Visualization of the mgm network follows the Fruchterman–Reingold algorithm (Fruchterman & Reingold, 1991) and was done via the *qgraph* and *bootnet* packages in R.

## Results

### Confirmatory Analyses

Bivariate Pearson correlations between all variables are presented in Table 3. Severity of IPV was associated with more severe childhood maltreatment, early maladaptive schemata (particularly mistrust), dissociation, BPD features, and PTSD symptoms, also when controlling for multiple comparisons and recruitment phase (before vs. after COVID-19 pandemic); see Table 4. Analyses for IPV perpetration and victimization separately revealed comparable results (see Appendixes B and C).

**Table 3.** Bivariate Pearson Correlations Between All Variables.

Variables	1	2	3	4	5	6	7	8	9	10	11	12	13	14	15	16	17
1. CTS2	—	.173^**^	.167^**^	.294^**^	−.067	.050	−.040	−.004	.040	.101^*^	.197^**^	−.054	.233^**^	.133^**^	.186^**^	.126^**^	.085
2. CTQ		—	.343^**^	.400^**^	−.048	.301^**^	.064	−.148^**^	.182^**^	.310^**^	.350^**^	−.462^**^	.346^**^	.357^**^	.237^**^	.316^**^	.279^**^
3. PAI-BOR			—	.477^**^	−.053	.459^**^	.144^**^	.075	.236^**^	.525^**^	.606^**^	−.163^**^	.596^**^	.481^**^	.430^**^	.441^**^	.525^**^
4. DES				—	−.160^**^	.441^**^	.133^**^	−.140^**^	.291^**^	.437^**^	.596^**^	−.240^**^	.438^**^	.393^**^	.236^**^	.435^**^	.371^**^
5. BERQ_AA					—	−.363^**^	.122^*^	.433^**^	−.343^**^	−.243^**^	−.199^**^	.247^**^	−.230^**^	−.201^**^	−.042	−.087	.039
6. BERQ_W						—	.145^**^	−.312^**^	.451^**^	.479^**^	.516^**^	−.290^**^	.552^**^	.530^**^	.295^**^	.410^**^	.355^**^
7. BERQ_SD							—	.075	.281^**^	.104^*^	.110^*^	−.015	.169^**^	.120^*^	.009	.058	.099^*^
8. BERQ_SS								—	−.342^**^	−.160^**^	−.075	.402^**^	−.161^**^	−.193^**^	.057	−.039	.145^**^
9. BERQ_Ign									—	.282^**^	.311^**^	−.250^**^	.340^**^	.268^**^	.083	.175^**^	.117^*^
10. AAS_Anxiety										—	.516^**^	−.357^**^	.655^**^	.446^**^	.325^**^	.453^**^	.383^**^
11. PCL											—	−.312^**^	.563^**^	.498^**^	.389^**^	.526^**^	.526^**^
12. MSPSS												—	−.355^**^	−.344^**^	−.145^**^	−.215^**^	−.088
13. YSQ Mistrust													—	.581^**^	.482^**^	.423^**^	.366^**^
14. YSQ Aleniation														—	.455^**^	.372^**^	.338^**^
15. YSQ Enmesh.															—	.290^**^	.310^**^
16. CERQ_SB																—	.597^**^
17. CERQ_RUM																	

*Note*. This table shows Pearson correlations. AAS = Adult Attachment Scale, attachment anxiety; CERQ = Cognitive Emotion Regulation Questionnaire; SB = self-blame; RUM = rumination; CTQ = Childhood Trauma Questionnaire; CTS2 = Conflict Tactics Scale revised; MSPSS = Multidimensional Scale of Perceived Social Support; BERQ = Behavioral Emotion Regulation Questionnaire; AA = actively approaching; W = withdrawal; SD = seeking distraction; SS = seeking social support, Ign = ignoring; PAI-BOR = Personality Assessment Inventory—Borderline Feature Scale; PCL = PTSD symptoms checklist; DES = Dissociative Experiences Scale; YSQ = Young Schema Questionnaire; Enmesh = enmeshment.

* *p* < .05. **p < .01.

**Table 4.** Separate Linear Regression Analyses Predicting IPV Perpetration and Victimization.

	*F*	*df*	*R²*	*R²*_adj_	*p*	
Model 1	6.70	2	.03	.03	.001*** ª	
Childhood maltreatment	*B*	*SE*	*β*	*t*	*p*	CI (95%)
CTQ sum	.344	.094	.172	3.66	<.001*** ª	[.160, .528]
Sample	−1.31	2.47	−.025	−.529	.597	[−6.172, 3.555]
Model 2	6.40	2	.03	.02	.002** ª	
BPD features	*B*	*SE*	*β*	*t*	*p*	CI (95%)
PAI-BOR total	.527	.150	.166	3.51	<.001*** ª	[.232, .823]
Sample	−1.22	2.49	−.023	−.490	.624	[−6,115, 3,674]
Model 3	20.13	2	.09	.09	<.001*** ª	
Dissociation	*B*	*SE*	*β*	*t*	*p*	CI (95%)
DES mean	6.06	.963	.294	6. 29	<.001*** ª	[4.16, 7.95]
Sample	1.95	2.49	.037	.784	.434	[−2.94, 6.84]
Model 4	0.72	6	.01	.01	.631	
Emotion regulation	*B*	*SE*	*β*	*t*	*p*	CI (95%)
BERQ actively approach.	−1.50	1.59	−.055	−.942	.347	[−4.63, 1.63]
BERQ withdrawal	1.23	1.65	.043	.748	.455	[−2.01, 4.48]
BERQ seeking distraction	-1.65	1.83	-.048	-.903	.367	[−5.26, 1.94]
BERQ seeking support	1.08	1.35	.045	.799	.425	[−1.58, 3.75]
BERQ ignoring	1.15	1.69	.041	.679	.497	[−2.18, 4.49]
Sample	1.00	2.60	.019	.386	.699	[−4.11, 6.1]
Model 5	2.47	3	.01	.01	.086	
Attachment	*B*	*SE*	*β*	*t*	*p*	CI (95%)
AAS-R anxiety	2.66	1.25	.102	2.14	.033*	[.216, 5.12]
Sample	1.55	2.50	.030	.622	.534	[−3.36, 6.48]
Model 6	8.94	2	.04	.04	<.001*** ª	
PTSD symptoms	*B*	*SE*	*β*	*t*	*p*	CI (95%)
PCL-20 total	.324	.078	.195	4.17	<.001*** ª	[.171, .477]
Sample	−1.01	2.47	−.019	−.407	.684	[−5.878, 3.859]
Model 7	0.86	2	.004	.001	.422	
Social support	*B*	*SE*	*β*	*t*	*p*	CI (95%)
MSPSS total	−.430	.396	−.052	−1.08	.279	[−1.209, .349]
Sample	−1.70	2.52	−.032	−.674	.500	[−6.667, 3.261]
Model 8	7.37	4	.07	. 06	<.001*** ª	
Early schemata	*B*	*SE*	*β*	*t*	*p*	CI (95%)
YSQ mistrust	4.539	1.436	.191	3.160	.002** ª	[1.72, 7.36]
YSQ alienation	−.490	1.156	−.025	−.424	.672	[−2.76, 1.78]
YSQ enmeshment	3.115	1.525	.113	2.042	.042*	[.117, 6.11]
Sample	−2.065	2.495	−.039	−.828	.408	[−6.97, 2.84]
Model 9	2.43	3	.02	.01	.065	
Cognitive coping	*B*	*SE*	*β*	*t*	*p*	CI (95%)
CERQ self-blame	3.581	1.782	.120	2.009	.045*	[.078, 7.08]
CERQ rumination	.321	1.854	.010	.173	.863	[−3.32, 3.97]
Sample	−1.428	2.545	−.027	−.561	.575	[−6.43, 3.58]

*Note*. AAS = Adult Attachment Scale, attachment anxiety; BERQ = Behavioral Emotion Regulation Questionnaire; BPD = borderline personality disorder; CERQ = Cognitive Emotion Regulation Questionnaire; CI = confidence interval; CTQ = Childhood Trauma Questionnaire; DES = Dissociative Experiences Scale; IPV = intimate partner violence; MSPSS = Multidimensional Scale of Perceived Social Support; PAI-BOR = Personality Assessment Inventory—Borderline Feature Scale; PCL = PTSD symptoms checklist; PTSD = posttraumatic stress disorder; YSQ = Young Schema Questionnaire.

ª Still significant after Bonferroni correction for the number of scales: *p* ≤ .003.

* *p* ≤ .05 (2-tailed). **p ≤ .01 (2-tailed). ***p ≤ .001 (2-tailed).

Results of the multiple regression analysis are summarized in Table 5. When including the other variables, childhood maltreatment severity still predicted IPV, while this correlation did not survive correction for multiple comparisons. IPV was still significantly linked to dissociation and mistrust.

**Table 5.** Multiple Linear Regression Analysis Predicting IPV Victimization and Perpetration.

IPV	*F*	*df*	*R²*	*R²*_adj_	*p*	
	4.68	17	.18	.14	<.001***	
Predictors	*B*	*SE*	*β*	*t*	*p*	CI (95%)
CTQ sum	.256	.119	.124	2.14	.033*	[−.049. .112]
PAI-BOR total	−.051	.233	−.015	−.218	.827	[−.031. .292]
DES mean	5.80	1.31	.284	4.40	.000***,ª	[.297. 2.17]
BERQ actively approaching	−2.16	1.60	−.077	−1.34	.178	[−1.50. .675]
BERQ withdrawal	−4.64	1.95	−.159	−2.38	.018*	[−2.44. .210]
BERQ seeking distraction	−2.28	1.81	−.065	−1.25	.209	[−1.18. 1.28]
BERQ seeking support	−.195	1.45	−.008	−.134	.893	[−1.48. .488]
BERQ ignoring	−.417	1.69	−.014	−.247	.805	[−.782. 1.50]
AAS-R attachment anxiety	−3.97	1.82	−.146	−2.17	.030*	[−2.60. −.133]
PCL total	.077	.126	.045	.610	.542	[−.101. .070]
MSPSS total	.695	.530	.080	1.312	.190	[−.659. .063]
YSQ mistrust	6.05	1.82	.245	3.307	.001***,ª	[−.384. .212]
YSQ alienation	−.424	1.32	−.021	−.320	.749	[−.871. .918]
YSQ enmeshment	3.38	1.63	.117	2.06	.039*	[.112. 2.33]
CERQ self-blame	−.070	1.97	−.002	−.035	.972	[−1.17. 1.51]
CERQ rumination	−1.59	2.07	−.051	−.769	.443	[−.518. 2.31]

*Note*. Controlling for sample (before vs. after start of COVID-19 pandemic, >0.05). AAS = Adult Attachment Scale, attachment anxiety; BERQ = Behavioral Emotion Regulation Questionnaire; CERQ = Cognitive Emotion Regulation Questionnaire; CI = confidence interval; CTQ = Childhood Trauma Questionnaire; DES = Dissociative Experiences Scale; IPV = intimate partner violence; MSPSS = Multidimensional Scale of Perceived Social Support; PAI-BOR = Personality Assessment Inventory—Borderline Feature Scale; PCL = PTSD symptoms checklist; PTSD = posttraumatic stress disorder; YSQ = Young Schema Questionnaire.

ª Still significant after Bonferroni correction for the number of scales: *p* ≤ .003.

* *p* < .05. ***p < .001 (2-tailed).

### Exploratory Network Analysis

**Figure 1.**
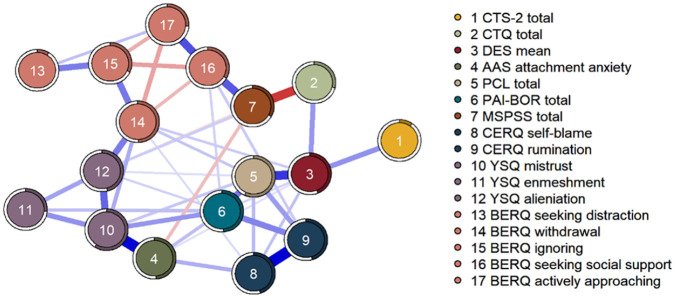
Network plot of the regularized MGM network. The thickness of a line indicates the strength of the association. Blue lines indicate positive associations and red lines indicate negative associations. The colored filled part of the ring around the nodes represents the predictability of the node by its connected neighbors (*R*^2^). MGM = mixed Gaussian model.

In the mgm network, 42 out of 136 possible edges (30.9%) were estimated to be non-zero (Figure 1). A correlation stability coefficient for node strength of .516 revealed that our network was sufficiently stable to be interpreted. Most central nodes were mistrust, withdrawal, PTSD symptoms, and BPD features. IPV was the least connected node in this network, which indicates its low centrality strength. IPV had only one unique (or direct) association with other variables, namely with dissociation. Dissociation further linked IPV to childhood maltreatment severity, as well as PTSD symptoms, and maladaptive coping (withdrawal and self-blame). More information on node strength and predictability, edge weights, bootstrapped difference tests, and confidence intervals can be found in Appendix D.

## Discussion

The aim of this study was to investigate associations between IPV and childhood maltreatment severity, early maladaptive schemata (mistrust, alienation, and enmeshment), anxious attachment, low social support, emotion dysregulation, dissociation, and symptoms of PTSD and BPD. By calculating a partial correlation network, we further explored the complex interplay of these variables, accounting for their mutual associations.

Rates of IPV victimization and perpetration were highly correlated, supporting the proposed reciprocal nature of IPV (Whitaker et al., 2007). Participants who were affected by the COVID-19 pandemic reported significantly more IPV victimization, even though this finding needs to be interpreted with caution, due to the small sample size. Largely in line with our hypothesis and previous research (see Li et al., 2019), we found a significant positive association between IPV and childhood maltreatment severity. Moreover, our study confirms previously observed positive associations between IPV and early maladaptive schemata (Pilkington et al., 2021), dissociation (Noll et al., 2003; Webermann & Brand, 2017; Webermann & Murphy, 2019; Zamir et al., 2018), PTSD symptoms (Birkley et al., 2016; Flanagan et al., 2014; Iverson et al., 2013; Sullivan et al., 2005), and BPD features (Krause-Utz et al., 2018, 2021). We further observed positive associations between IPV and attachment anxiety as well as maladaptive emotion regulation strategies (self-blame, withdrawal), which is in line with earlier findings (Batten et al., 2001; Bonache et al., 2017; Kong et al., 2018; Lawson & Brossart, 2013; McKeown, 2014; Roth & Cohen, 1986; Scoglio et al., 2021). These findings need to be interpreted with caution, however, since they did not survive correction for multiple comparisons, possibly due to a lack of statistical power. Discrepancies with earlier findings may also partly be explained by the selection of our scales, since we focused on attachment anxiety as well as specific coping strategies. Future studies should elucidate the role of other attachment styles (e.g., avoidant attachment) and underlying emotion regulation difficulties (e.g., identifying and labeling emotions, emotional instability, and reactivity) as well as alcohol consumption in this context. Future studies may also focus on specific schema modes to gain deeper insights. No significant association was observed with social support, which has been previously implicated in IPV (Capaldi et al., 2012; Scoglio et al., 2021). A possible explanation for this is that we assessed perceived social support, which may not necessarily reflect objective support.

In an exploratory, hypothesis-generating manner, we explored the overall interplay of all variables using graph-theoretical network analysis. In this analysis, IPV was not linked to childhood maltreatment severity anymore, but only indirectly via dissociation. Interestingly, dissociation was also the only outcome that was still uniquely linked to IPV in this partial correlation network. Dissociation was further associated with PTSD symptoms and maladaptive emotion regulation strategies (ignoring, withdrawal, and rumination). This is line with conceptualizations of dissociation as a maladaptive coping response to escape unpleasant or traumatic experiences (Lanius et al., 2010). Dissociation can develop very early in life, when stressful circumstances (e.g., severe and ongoing abuse or neglect by primary caregivers) exceed the coping resources of an individual (Dorahy et al., 2016; Vonderlin et al., 2018). Persistent dissociative symptoms, such as de-personalization, de-realization, and memory fragmentation, can create an inner distance from abusive experiences that are beyond control. In the long run, however, this interferes with learning, memory, and problem-solving processes (Lanius et al., 2010). In abusive partner relationships, dissociation may dampen information processing, lower perceived control and assertiveness, and thereby enhance the risk of revictimization ((Noll et al., 2003; Zamir et al., 2018; Zurbriggen & Freyd, 2004). At the same time, dissociation may also promote IPV perpetration, as violent acts may feel disconnected and out of control (Webermann & Brand, 2017; Webermann & Murphy, 2019). In the context of earlier prospective and observational studies, our findings point to an important role of dissociation in IPV. Dissociative responses should be closely monitored and targeted in individuals, who experience interpersonal violence.

To our knowledge, this study is the first to investigate various psychological correlates of IPV simultaneously to account for their multivariate interplay. More research is needed to understand the complex interplay of these factors. In particular, further research with prospective data is needed to better elucidate the psychological mechanisms underlying the relation between childhood maltreatment and IPV, with respect to early maladaptive schemata, attachment, social support, dissociation, and symptoms of PTSD and BPD.

Due to the cross-correlational nature of our design, the interpretation of possible causal directions is limited. Longitudinal studies that compare incidents of family and partner violence across a longer time period will help to understand causal pathways. Moreover, findings need to be interpreted with caution and corroborated by further research, due to the relatively small sample size. Future studies should also take situational partner dynamics and other contextual factors (e.g., cultural environment and financial situation) into account (Messman-Moore & Long, 2003, Whitaker et al., 2007). Diversity was addressed by posting the anonymous online survey on mental health platforms that were accessible to individuals with a wide range of age, gender, socioeconomic status, race, ethnicity, and culture. We included individuals from different nationalities, although one inclusion criterion was sufficient English proficiency. Results of our study may therefore not be generalized to those lacking English proficiency. More research with a stronger gender balance and acknowledging other aspects of diversity is needed to extend our findings. While IPV seems to be more common in younger populations (Bonache et al., 2017; Karakurt & Silver, 2013), research in older populations is needed to understand IPV across the lifespan.

In conclusion, our findings suggest that IPV perpetration and victimization often co-occur. When accounting for all variables in one model, IPV was uniquely connected to dissociation, which may be an important bridge symptom, linking it to childhood maltreatment experiences, PTSD symptoms, and maladaptive coping. More research is needed to understand direct and indirect psychological pathways in the cycle of violence.

## Supplemental Material

sj-docx-1-jiv-10.1177_08862605231181377 – Supplemental material for Psychological Factors Linked to Intimate Partner Violence and Childhood Maltreatment: On Dissociation as a Possible Bridge Symptom Click here for additional data file. Supplemental material, sj-docx-1-jiv-10.1177_08862605231181377 for Psychological Factors Linked to Intimate Partner Violence and Childhood Maltreatment: On Dissociation as a Possible Bridge Symptom by Annegret Krause-Utz, Romana Černáková, William Hoogenboom, Anna Schulze, Sarah Büttner, Zeynep Demirelli, Joanne Mouthaan, Charlotte C. van Schie, Nadia Garnefski and Vivian Kraaij in Journal of Interpersonal Violence

sj-docx-2-jiv-10.1177_08862605231181377 – Supplemental material for Psychological Factors Linked to Intimate Partner Violence and Childhood Maltreatment: On Dissociation as a Possible Bridge Symptom Click here for additional data file. Supplemental material, sj-docx-2-jiv-10.1177_08862605231181377 for Psychological Factors Linked to Intimate Partner Violence and Childhood Maltreatment: On Dissociation as a Possible Bridge Symptom by Annegret Krause-Utz, Romana Černáková, William Hoogenboom, Anna Schulze, Sarah Büttner, Zeynep Demirelli, Joanne Mouthaan, Charlotte C. van Schie, Nadia Garnefski and Vivian Kraaij in Journal of Interpersonal Violence

sj-docx-3-jiv-10.1177_08862605231181377 – Supplemental material for Psychological Factors Linked to Intimate Partner Violence and Childhood Maltreatment: On Dissociation as a Possible Bridge Symptom Click here for additional data file. Supplemental material, sj-docx-3-jiv-10.1177_08862605231181377 for Psychological Factors Linked to Intimate Partner Violence and Childhood Maltreatment: On Dissociation as a Possible Bridge Symptom by Annegret Krause-Utz, Romana Černáková, William Hoogenboom, Anna Schulze, Sarah Büttner, Zeynep Demirelli, Joanne Mouthaan, Charlotte C. van Schie, Nadia Garnefski and Vivian Kraaij in Journal of Interpersonal Violence

sj-docx-4-jiv-10.1177_08862605231181377 – Supplemental material for Psychological Factors Linked to Intimate Partner Violence and Childhood Maltreatment: On Dissociation as a Possible Bridge Symptom Click here for additional data file. Supplemental material, sj-docx-4-jiv-10.1177_08862605231181377 for Psychological Factors Linked to Intimate Partner Violence and Childhood Maltreatment: On Dissociation as a Possible Bridge Symptom by Annegret Krause-Utz, Romana Černáková, William Hoogenboom, Anna Schulze, Sarah Büttner, Zeynep Demirelli, Joanne Mouthaan, Charlotte C. van Schie, Nadia Garnefski and Vivian Kraaij in Journal of Interpersonal Violence
