# Supplementary material for: Psychological Factors Linked to Intimate Partner Violence and Childhood Maltreatment: On Dissociation as a Possible Bridge Symptom
Source: J Interpers Violence. 2023 Jul 11;38(21-22):11400–28. doi: 10.1177/08862605231181377 (PMC10515471; doi:10.1177/08862605231181377)
Supplement: sj-docx-1-jiv-10.1177_08862605231181377 – Supplemental material for Psychological Factors Linked to Intimate Partner Violence and Childhood Maltreatment: On Dissociation as a Possible Bridge Symptom [file sj-docx-1-jiv-10.1177_08862605231181377.docx]

| **Appendix A** |  |  |  |
| --- | --- | --- | --- |
|  | Sample 1  Before pandemic | Sample 2  N= 139  After pandemic | Group statistics  (χ², df, p) |
| *Gender*  Female  Male | *n=* 190 (70%)  *n*= 77 (28%) | *n*= 54 (39%)*  *n*= 51 (37%)* | χ²_(4)_= 68.06,  p < .001 |
| *Education*  Primary School  Secondary School  Bachelor Degree  Master Degree  PhD  Vocational Training  Other | *n* =1 (0.4%)  *n* = 160 (59%)  *n* = 63 (23%)  *n* = 29 (11%)  *n* = 3 (1%)  *n* = 9 (3%)  *n* = 6 (2%) | *n =* 1 (0.7%)  *n =* 76 (55%)  *n =* 34 (24%)  *n =* 18 (13%)  *n =* 3 (2%)  *n =* 2 (1%)  *n =* 5 (4%) | χ²_(6)_ = 3.56,  p = .74 |
| *Relationship Status*  Single  In a relationship  Married  Divorced  Separated  Widowed  Shared household  Other | *n* = 104(38%)  *n* = 104(38%)  *n* = 28 (10%)  *n* = 0 (0%)  *n* = 1 (0.5%)  *n* = 1 (0.5%)  *n* = 3 (1%)  *n* = 4 (2%) | *n =* 49 (35%)  *n =* 58 (42%)  *n =* 18 (7%)  *n =* 2 (1%)  *n =* 0 (0%)  *n =* 1 (0.7%)  *n =* 4 (3%)  *n =* 0 (0%) | χ²_(8)_=11.82,  p = .16 |
| *Nationality*  European  Asian  North American  South American  Middle East  Other | *n* = 242(89%)  *n* = 8 (3%)  *n* = 6 (2%)  *n* = 4 (1.5%)  *n* = 4 (1.5%)  *n* = 7 (2.5%) | *n =* 100(72%)  *n =* 5 (4%)  *n =* 0 (0%)  *n =* 0 (0%)  *n =* 29 (21%)  *n =* 5 (4%) | *χ²_(5)_= 51.80,*  *p < .001* |
| *Currently in education*  Yes  No | *n* = 220 (81%)  *n* = 51 (19%) | *n =* 105(76%)  *n =* 34 (24%) | χ²_(1)_= 1.78,  p = .18 |
| *Currently employed*  Yes  No | *n* =150(55%)*  *n* =120(45%)* | *n =* 66(48%)*  *n =* 72(52%)* | χ²_(1)_= 2.19,  p = .14 |
| *Currently in treatment*  Yes  No | *n =* 28(10%)*  *n=*242(90%)* | *n =* 22 (16%)  *n =* 117 (84%) | χ²_(1)_= 2.55,  p = .11 |
| *Previous treatment*  Yes  No | *n=*100(37%)*  *n=*170(63%)* | *N =* 61 (44%)  *n =* 78 (56%) | χ²_(1)_= 1.80,  p = .18 |

* Missing data: N_S1_= 270; N_S2_= 138
