# Supplementary material for: Psychological Factors Linked to Intimate Partner Violence and Childhood Maltreatment: On Dissociation as a Possible Bridge Symptom
Source: J Interpers Violence. 2023 Jul 11;38(21-22):11400–28. doi: 10.1177/08862605231181377 (PMC10515471; doi:10.1177/08862605231181377)
Supplement: sj-docx-3-jiv-10.1177_08862605231181377 – Supplemental material for Psychological Factors Linked to Intimate Partner Violence and Childhood Maltreatment: On Dissociation as a Possible Bridge Symptom [file sj-docx-3-jiv-10.1177_08862605231181377.docx]

**Appendix C**:

*Separate linear regression analyses predicting IPV Victimization*

| **Model 1 CTQ** | ***F*** | ***df*** | ***R²*** | ***R²_adj_*** | ***p*** |  |
| --- | --- | --- | --- | --- | --- | --- |
| *Child maltreatment* | 2.59 | 6 | .03 | .02 | .018 | * |
| **Model 2 PAI BOR** | ***F*** | ***df*** | ***R²*** | ***R²_adj_*** | ***p*** |  |
| *BPD features* | 2.61 | 5 | .03 | .02 | .024 | * |
| **Model 3 DES** | ***F*** | ***df*** | ***R²*** | ***R²_adj_*** | ***p*** |  |
| *Dissociation* | 21.88 | 2 | .10 | .09 | <.001 | *** |
| **Model 4 BERQ** | ***F*** | ***df*** | ***R²*** | ***R²_adj_*** | ***p*** |  |
| *Emotion regulation* | 0.49 | 6 | .01 | .01 | .819 |  |
| **Model 5 AAS-R** | ***F*** | ***df*** | ***R²*** | ***R²_adj_*** | ***p*** |  |
| *Attachment* | 2.62 | 2 | .01 | .01 | .074 |  |
| **Model 6 PCL** | ***F*** | ***df*** | ***R²*** | ***R²_adj_*** | ***p*** |  |
| *PTSD symptoms* | 5.80 | 5 | 0.06 | 0.05 | <.001 | *** |
| **Model 7 MSPSS** | ***F*** | ***df*** | ***R²*** | ***R²_adj_*** | ***p*** |  |
| *Social support* | 0.59 | 4 | 0.01 | 0.004 | .668 |  |
| Predictors | *B* | *SE* | *β* | *t* | *p* |  |
| **Model 8 YSQ** | ***F*** | ***df*** | ***R²*** | ***R²_adj_*** | ***p*** |  |
| *Early schemata* | 6.00 | 4 | .05 | .04 | <.001 | *** |
| **Model 9 CERQ** | ***F*** | ***df*** | ***R²*** | ***R²_adj_*** | ***p*** |  |
| *Cognitive coping* | 1.91 | 3 | .01 | .01 | .172 |  |

Note: * p ≤ .05 (2-tailed); ** p ≤ .01 (2- tailed). *** p ≤ .001 (2- tailed)
