# Supplementary material for: Psychological Factors Linked to Intimate Partner Violence and Childhood Maltreatment: On Dissociation as a Possible Bridge Symptom
Source: J Interpers Violence. 2023 Jul 11;38(21-22):11400–28. doi: 10.1177/08862605231181377 (PMC10515471; doi:10.1177/08862605231181377)
Supplement: sj-docx-4-jiv-10.1177_08862605231181377 – Supplemental material for Psychological Factors Linked to Intimate Partner Violence and Childhood Maltreatment: On Dissociation as a Possible Bridge Symptom [file sj-docx-4-jiv-10.1177_08862605231181377.docx]

**Appendix D: detailed information of the mgm Network**

**Table S1**

*Centrality indices and predictability of nodes in network*

| node | centrality strength | predictability R^2^ |
| --- | --- | --- |
| CTS-2 total | .149 | .098 |
| CTQ total | .487 | .292 |
| DES mean | .830 | .439 |
| AAS attachment anxiety | .677 | .489 |
| PCL total | .902 | .569 |
| PAI-BOR total | .873 | .490 |
| MSPSS total | .655 | .333 |
| CERQ self-blame | .694 | .451 |
| CERQ rumination | .729 | .454 |
| YSQ mistrust | 1.044 | .594 |
| YSQ enmeshment | .367 | .266 |
| YSQ alienation | .762 | .460 |
| BERQ seeking distraction | .266 | .089 |
| BERQ withdrawal | .908 | .495 |
| BERQ ignoring | .608 | .319 |
| BERQ seeking social support | .838 | .333 |
| BERQ actively approaching | .566 | .263 |

**Table S2**

*Edge weights in mgm network*

|  | 1 | 2 | 3 | 4 | 5 | 6 | 7 | 8 | 9 | 10 | 11 | 12 | 13 | 14 | 15 | 16 |
| --- | --- | --- | --- | --- | --- | --- | --- | --- | --- | --- | --- | --- | --- | --- | --- | --- |
| 1 CTS-2 total |  |  |  |  |  |  |  |  |  |  |  |  |  |  |  |  |
| 2 CTQ total | 0 |  |  |  |  |  |  |  |  |  |  |  |  |  |  |  |
| 3 DES mean | .149 | .143 |  |  |  |  |  |  |  |  |  |  |  |  |  |  |
| 4 AAS attachment anxiety | 0 | 0 | .042 |  |  |  |  |  |  |  |  |  |  |  |  |  |
| 5 PCL total | 0 | 0 | .275 | 0 |  |  |  |  |  |  |  |  |  |  |  |  |
| 6 PAI-BOR total | 0 | 0 | .063 | .090 | .190 |  |  |  |  |  |  |  |  |  |  |  |
| 7 MSPSS total | 0 | -.275 | 0 | -.090 | 0 | 0 |  |  |  |  |  |  |  |  |  |  |
| 8 CERQ self-blame | 0 | 0 | .087 | .111 | .094 | 0 | 0 |  |  |  |  |  |  |  |  |  |
| 9 CERQ rumination | 0 | 0 | 0 | 0 | .136 | .171 | 0 | .351 |  |  |  |  |  |  |  |  |
| 10 YSQ mistrust | 0 | 0 | 0 | .344 | .078 | .144 | 0 | 0 | 0 |  |  |  |  |  |  |  |
| 11 YSQ enmeshment | 0 | 0 | 0 | 0 | 0 | .089 | 0 | 0 | 0 | .150 |  |  |  |  |  |  |
| 12 YSQ alienation | 0 | .069 | 0 | 0 | .049 | .064 | -.059 | 0 | 0 | .213 | .128 |  |  |  |  |  |
| 13 BERQ seeking distraction | 0 | 0 | 0 | 0 | 0 | 0 | 0 | 0 | 0 | 0 | 0 | 0 |  |  |  |  |
| 14 BERQ withdrawal | 0 | 0 | .071 | 0 | .080 | 0 | 0 | .052 | 0 | .114 | 0 | .180 | 0 |  |  |  |
| 15 BERQ ignoring | 0 | 0 | 0 | 0 | 0 | 0 | 0 | 0 | 0 | 0 | 0 | 0 | .180 | .187 |  |  |
| 16 BERQ seeking social support | 0 | 0 | 0 | 0 | 0 | .063 | .230 | 0 | .072 | 0 | 0 | 0 | 0 | -.097 | -.132 |  |
| 17 BERQ actively approaching | 0 | 0 | 0 | 0 | 0 | 0 | 0 | 0 | 0 | 0 | 0 | 0 | .086 | -.127 | -.109 | .244 |

**
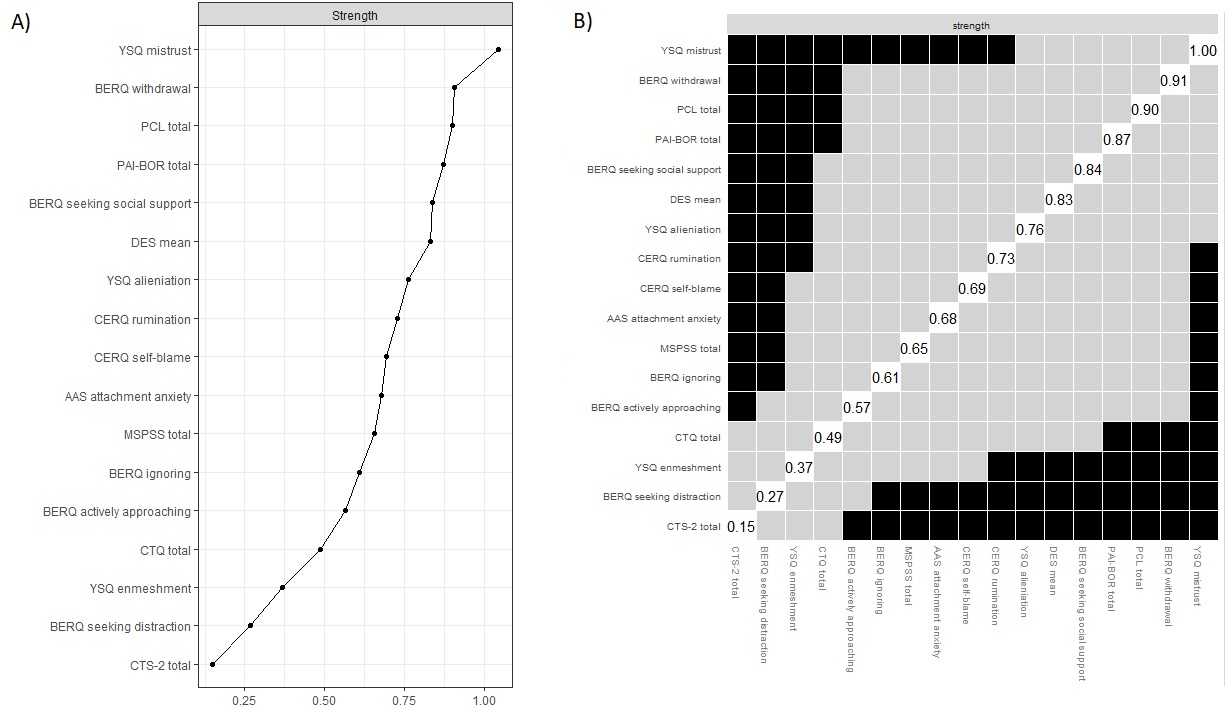
**

**Figure S1.** A) Nodes strength and B) bootstrapped difference test on node strength.


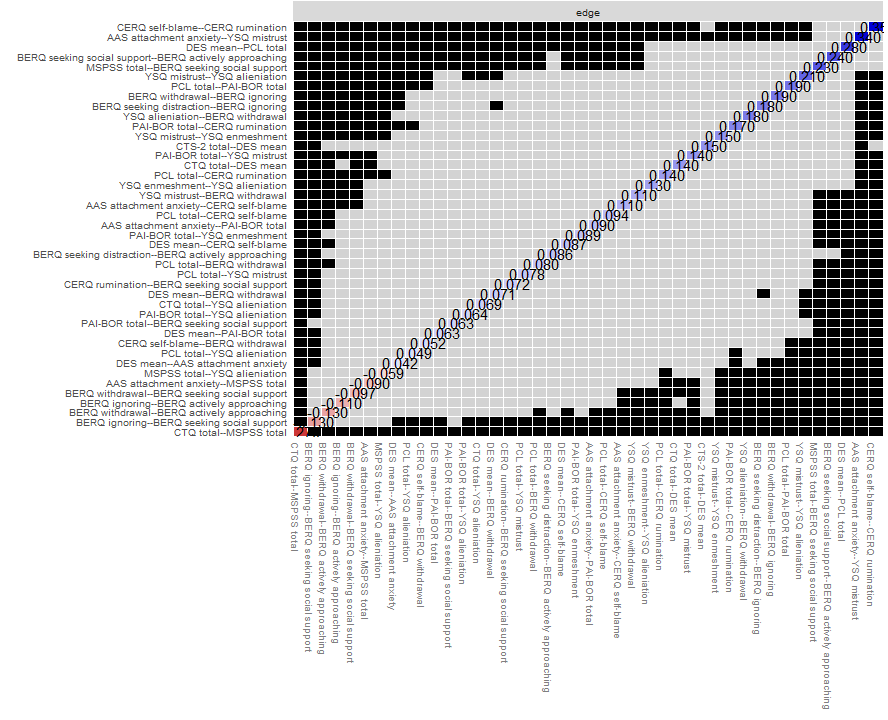


**Figure S2.** Bootstrapped difference test on edge weigths.

**
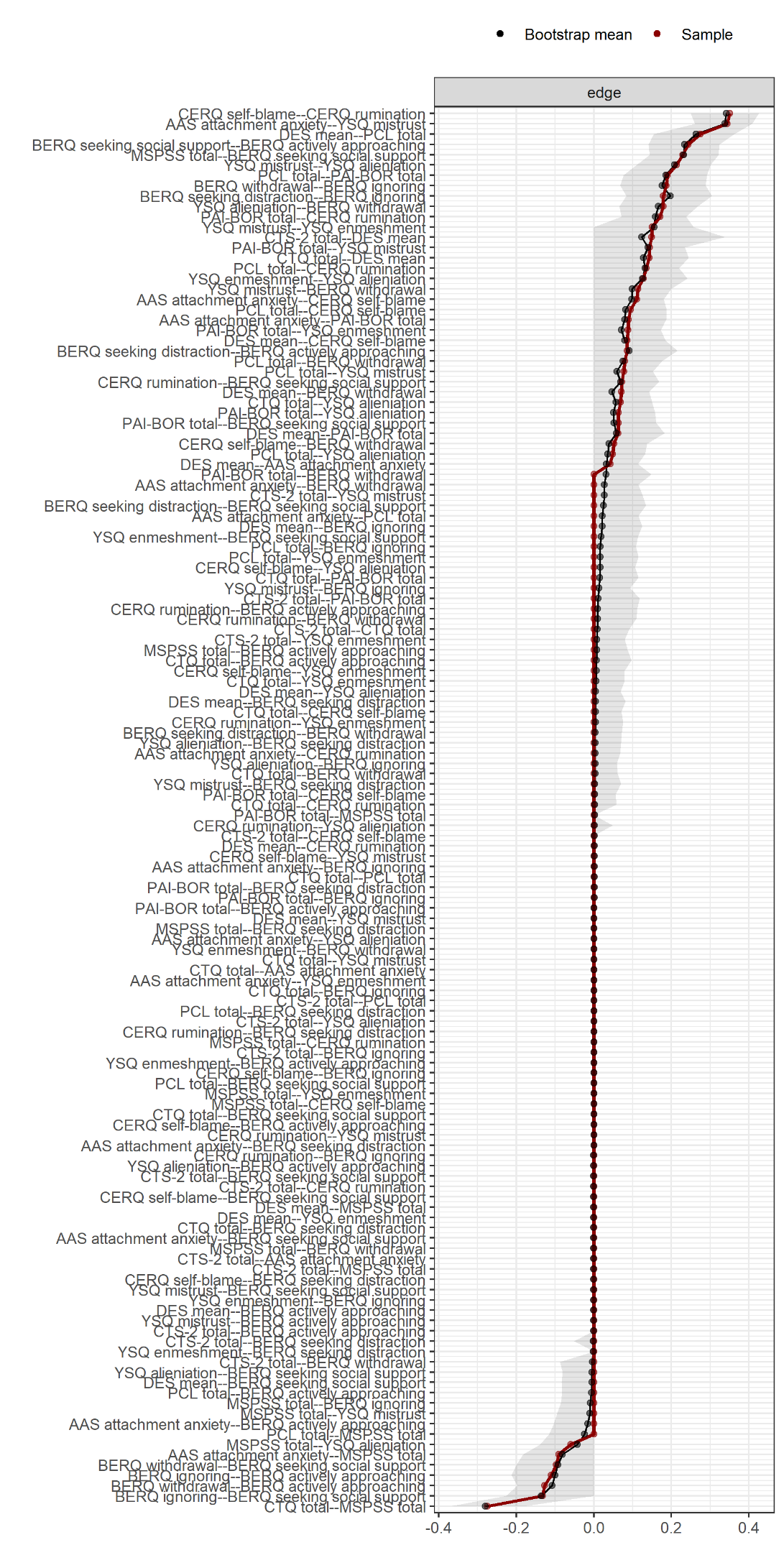
**

**Figure S3.** Bootstrapped confidence intervals of edge weigths.
